# Challenges in understanding inequities in help-seeking for possible cancer symptoms

**DOI:** 10.1186/s44263-024-00082-1

**Published:** 2024-07-18

**Authors:** Katriina L. Whitaker, Tetyana Perchyk, Robert S. Kerrison, Agnieszka Lemanska

**Affiliations:** 1 Faculty of Health and Medical Sciences, University of Surrey, Guildford, UK; 2 Data Science Department, National Physical Laboratory, Teddington, UK

**Keywords:** Inequities, Help-seeking, Cancer, Early diagnosis

## Abstract

Tackling inequities in cancer outcomes is a global health priority. One avenue for improving early diagnosis of cancer is to ensure people know when and how to seek help for cancer symptoms and that this knowledge (and behaviour) is equitably distributed across the population. In this perspective piece we highlight the challenges in understanding sociodemographic differences in help-seeking behaviour (for example, how help-seeking is defined / conceptualised and subsequently assessed), as well as challenges with using existing datasets that are now more readily accessible than ever. Addressing these will strengthen methodological approaches to understand inequities in help-seeking and ways to tackle them.

## Background

Public health campaigns designed to prompt help-seeking for possible cancer symptoms help raise awareness of signs and symptoms and/or tackle barriers such as fear of cancer. Evidence suggests that campaigns increase awareness, help-seeking in primary care, and urgent primary care referrals [1] and this has showed promise in shifting the stage distribution towards earlier stages of specific cancers (e.g. lung cancer [2, 3]; bladder cancer [4]). Yet there is evidence that these campaigns do not have the same influence across different sociodemographic groups [5]. It is possible to tailor campaigns to avoid exacerbating inequities in cancer outcomes, for example by targeting those who are less aware of symptoms, or less likely to seek help [6]. However, this requires an understanding of where and why differences exist.

In this perspective piece, we discuss challenges in understanding inequities in help-seeking, including conceptualising help-seeking and data challenges. We also consider evidence for inequities in help-seeking according to different sociodemographic groups, including ethnic minority communities, people with a learning disability, people with multiple long-term health conditions, and other groups that share protected characteristics (e.g. age, gender). We use the term inequity to infer that there are differences, which are unnecessary and avoidable and can be considered unfair and unjust [7].

## Challenges with help-seeking and inequities research

### Conceptualising help-seeking

The Model of the Pathways to Treatment defines help-seeking as the interval between perceiving a reason to discuss a symptom with a healthcare professional and the first consultation with a healthcare professional about that symptom [8]. While this definition has been widely adopted and integrated with psychological theory (e.g. the Common Sense Model of Illness [9]), a key ongoing challenge with help-seeking research is how to measure it.

Historically, measures such as the Cancer Awareness Measure [10] and the ABC measure [11], have measured help-seeking intentions (e.g. *if you noticed a symptom, how soon would you contact the doctor about it?*), as opposed to behaviour in response to real life symptom experiences. A large systematic review found that studies using hypothetical measures typically report shorter time intervals than studies asking about real life help-seeking experiences [12]. This difference is often referred to as the ‘intention-behaviour gap’ or ‘hypothetical bias’ [13]. Importantly, this difference in approach influences reported associations between socioeconomic group and help-seeking, with studies measuring actual time to presentation reporting longer delays among individuals from lower socioeconomic backgrounds (compared to those from higher socioeconomic backgrounds) than those using anticipated or hypothetical help-seeking [12]. It is not clear why there are differences (e.g. these could relate to differences in social desirability bias), but the key message is that at a minimum we should be measuring actual, as opposed to anticipated help-seeking. In line with this, the most recent versions of the Cancer Awareness Measure now assess actual help-seeking [10].

There are further challenges with measuring help-seeking behaviour that should be recognised when drawing conclusions about inequity in peoples’ responses. For example, although self-reported help-seeking (yes/no) shows good correspondence with primary care records [14], accurately measuring time intervals (number of days) is more challenging. Even defining what is meant by “shorter” versus “longer” help-seeking intervals is difficult, because this is symptom/disease specific; waiting two or more weeks to report a persistent cough or weight loss may not be considered the same as waiting to report coughing up blood or a breast lump. This has resulted in many studies using an oversimplified dichotomous help-seeking variable (i.e. sought help in last six months and have not sought help) instead [15], losing potentially helpful detail about timeliness (and, subsequently, sociodemographic variation in timeliness) of presentation.

Another challenge involves capturing the complexity of help-seeking, which in reality is rarely a one-off behaviour/event but an ongoing interaction between people and the healthcare system, which can be influenced by previous help-seeking experiences [16]. It is therefore important to consider help-seeking as a broader range of behaviours/ experiences than simply asking about if/ when someone has contacted their doctor or healthcare provider about a symptom. Proxies for help-seeking behaviour are already widely used (e.g. perceived barriers to presenting to primary care with symptoms) and a significant proportion of studies reporting on socio-demographic variation (particularly ethnic differences) describe antecedents to behaviour (e.g. knowledge, beliefs, attitudes) and/or barriers to help-seeking [17–20].

A useful framework to further help conceptualise the complexity of help-seeking is the candidacy framework [21], which moves away from measures related to utilisation (e.g. number of consultations) because of assumptions about what is considered normative or acceptable. Instead, the candidacy framework captures the complex interplay between several processes involving people, healthcare services and wider contextual factors within seven key elements. These include identification of need (how people recognise symptoms need medical attention), navigation (awareness of/attendance at services), permeability of services (ease of use), appearance at services (asserting a claim for need), adjudications (professional judgement of need), offers and resistance (e.g. refusal of services) and operating conditions and local production of candidacy (e.g. availability/suitability of resources) [21].

In traditional help-seeking research, the focus is usually on the first two elements (identification and navigation), meaning important information may be lost about potential differences/ inequities within other dimensions. Further validated measures are required to capture constructs related to perceived eligibility for accessing and re-accessing healthcare [22]. In accordance with this, the Blood Cancer Awareness measure was designed to assess a wider array of factors, including patient enablement and reconsultation (as well as help-seeking) behaviour. This has shown some early promise in terms of being able to delineate relationships between patient factors and help-seeking [23], but further work is required to understand the implications for evidence on inequities in help-seeking. Another limitation is that most cancer awareness measures have been developed in the UK (e.g. CAM, Blood CAM), although these have been adapted to be applied in different context (e.g. ABC measure) [24], it is crucial that we maintain a global lens on understanding inequities in help-seeking.

It is also important to consider the changing landscape of help-seeking in primary care – this is well captured by a new theory of digital candidacy, which brings together elements of the candidacy framework with theories related to socio-technical and technology structuration, which explore the need to design technology to account for the needs and diversity of its users [25]. One of the central arguments is that patients increasingly need to create a digital facsimile (a digital version of themselves / their concerns for example, via online triaging systems) to articulate need before adjudication, and some people may be able to do this more easily than others.

### Challenges in using routinely collected data

Complete and reliable data are a fundamental resource to aid understanding of where inequities in help-seeking exist [26, 27]. Healthcare commissioners and providers have an obligation to collect good quality, timely data, and these data should be used to identify inequities and to promptly act on them. Clinical audits are well established tools used to understand the shortfalls in healthcare. However, they are rarely used to assess differences between diverse patient groups. The Healthcare Quality Improvement Partnership (HQIP), who manage the UK’s largest clinical audits, published a report in 2020 on addressing health inequality in national audits [28]. In the report, they demonstrated that patients’ characteristics, such as age, sex, geographical location, and ethnicity, were widely recorded in national audits. However, characteristics such as disability (including learning disability) and mental illness, which can also affect help-seeking, were rarely recorded or not recorded at all.

The report by the Australian Institute of Health and Welfare (AIHW, 2018) highlighted similar gaps in data quality in national surveys and administrative datasets in Australia [29]. These were related to the limited capture of individual characteristics such as ethnicity, sexuality, disability and mental health status. The report led to a call in 2022 for improved Australian data on social determinants of health inequities [30], including the need for a nationally agreed, consistent disability identifier [31]. Similar issues were found in the US, with standards for health data published in 2022 by the Centers for Disease Control and Prevention (CDC) [32], and a review of the state of health disparities indicating underrepresentation and limited generalisability of population-level studies for racial and ethnic minorities [33].

Observational studies that use routinely collected healthcare records are similarly affected by these issues, although there are methods that can be used to curate patient characteristics from healthcare information. For example, in the absence of patient-level deprivation or socioeconomic status (SES), these variables are often curated from patients’ geographical locations or postcodes of their healthcare providers [34, 35]. Similarly, when extracting ethnicity, algorithms based on available demographic information such as self-reported ethnicity, country of origin and language are used. However, these curated patient characteristics are highly affected by a large proportion of missing data, uncertainty in data quality and reliability, as well as a lack of standardised terminology. This is in part because healthcare data are not primarily designed for research.

Another challenge for observational studies in investigating help-seeking inequities is related to the analytical protocols to handle missing data. They are usually based on excluding participants with missing data. This approach, called complete case analysis, can produce bias in results, which perpetuates the inequities further [36]. Sensitivity analyses, aimed at reducing this bias, often use multiple imputation methods which require that patients’ characteristics data are missing at random [37]. This assumption is violated by the inherent quality of data associated with inequities, limiting interpretability and useability of the results. The relationship between inequities and help-seeking is complex because there are many interrelated factors that affect it. Any meaningful utilisation of healthcare data in this area requires sensitive knowledge and experience. This source of complexity requires advanced statistical approaches capable of unravelling causal effect from correlated variables that are susceptible to confounding, as well as mixed methods approaches that help corroborate the results [38]. When designing a study, careful consideration should be given to planning which factors to include, what the complex interactions are, understanding the suitability of methodological approaches and limitations of data, and accounting for uncertainty due to missing data.

The power of observational studies for understanding inequities lies in observing real-world scenarios. The COVID-19 pandemic, which challenged nearly every sector of healthcare, has left an important legacy. As a rapid response was critical, the pandemic has significantly propelled the growth of healthcare analytics in the UK and worldwide [39]. Large datasets, some with 100% coverage of the populations, can now be accessed, raising opportunities for research. In the UK, these promising infrastructures include the NHS England Secure Data Environment (https://digital.nhs.uk/services/secure-data-environment-service), the Office for National Statistics Secure Research Service (https://integrateddataservice.gov.uk/) and University of Oxford’s OpenSAFELY (https://www.opensafely.org/). These trusted research environments (TRE) are of unprecedented size and quality and hold data which should be utilized to drive improvements in healthcare inequalities. However, it is important to note that, despite the increasing size and quality of datasets, the challenges related to limitations of the curated patient characteristics remain relevant.

Healthcare providers and researchers alike are mandated to use these data for patient benefit. Due to concerns surrounding privacy and confidentiality, data controllers had previously been reluctant to share personal data. TREs have addressed this issue by closely working within the boundaries of what is acceptable to patients and clinicians [40–42], greatly increasing the availability of healthcare data for research. As a consequence, we have never been in a better position to deliver inclusive research with the aim to improve inequities [43].

However, this increased access to data must be underpinned by ongoing collaborations between patients, stakeholders and researchers, to ensure public trust and confidence that their data will be used ethically, transparently and securely.

In the UK, patients can opt-out of their data being used in research. Poorly run data sharing campaigns which fail to adequately address ethical concerns, transparency issues and meaningful stakeholder engagement can lead to damaging erosion in public support [44]. Some of the more notable examples in the UK include the care.data programme in 2016 [45], the General Practice Data for Planning and Research scheme in 2021 [46], and more recently the Federated Data Platform introduced in 2023 [47]. They all faced criticism for failing to address the fundamental building blocks of public trust, which resulted in scepticism linked to waves of people opting out, and eventually the abandonment of the initiatives altogether.

According to a dashboard run by the NHS (https://digital.nhs.uk/dashboards/national-data-opt-out-open-data), currently there are over 5% of people with an opt-out status and this varies by age, sex and region [48]. Apart from the more obvious reductions in sample sizes for observational studies, the consequences of opt-outs for research are not yet fully clear. However, with differences in opt-out rates between different groups of people, limited generalisability, bias and perpetuation of inequalities are all plausible outcomes. Therefore, improving public awareness and engagement, as well as ensuring safety and confidentiality, are key safeguards required to mitigate the potential negative consequences.

## Evidence for inequities in help-seeking across socio-demographic groups

Against a backdrop of challenges there has been a substantial amount of work to understand inequities in help-seeking. This has shown that for SES and comorbidities, there is mixed evidence for influences on help-seeking; lower SES and having a comorbidity have been associated with both shorter and longer help-seeking intervals [12, 49–51]. However, for several other characteristics, there is a lack of evidence to support the notion that they are associated with help-seeking at all, partly because there has been very little research (e.g. for gender, learning disability) [52–54]. The only sociodemographic variable where there is consistent evidence appears to be for age, with older age usually associated with prompter help-seeking [55].

A number of explanatory factors have been proposed to explain differences in help-seeking behaviour, but studies rarely explore the links between sociodemographic characteristics, potential mediators and real-life help-seeking behaviour. These factors are often broadly divided into cognitive (e.g. knowledge of symptoms), emotional (e.g. fear, fatalism), practical (e.g. being too busy, financial challenges) or healthcare system factors (e.g. trust in the system, challenges with access). There are also other variables that have so far been underexplored in research particularly since the pandemic and the potential exacerbation of issues around public trust and access to services [56].

Broadening our view of potential influences on help-seeking behaviours and how they intersect is critical to ensure we generate an authentic understanding of the issues that need to be addressed. Although analyses often control for other potentially confounding variables (e.g. age), less attention is paid to the intersections between variables and how it is likely that a combination of socio-demographic characteristics influence help-seeking behaviour. Intersectionality can provide a critical lens to understand how the interconnectedness of multiple identities and social processes influence cancer outcomes and experiences [57]. An example outside of the cancer literature demonstrates this point by exploring the intersection of age and living in rural communities in the context of healthcare seeking more generally [58]. They reported that multiple factors such as education level, gender, and socioeconomic status were associated with help-seeking behaviour in older rural communities. The recent Women, power and cancer Lancet Commission [59] also highlighted a myriad of factors and overlapping forms of discrimination (e.g. age, ethnicity, socioeconomic status, gender identity) that are likely to challenge access to early detection and diagnosis of cancer for women. Researchers need to become equipped to analyse the ways that multiple forms of disadvantage influence experiences of disenfranchisement [60], including help-seeking experiences.

## Conclusions

In this perspective piece, we described a number of challenges conceptualising help-seeking and the analysis of help seeking behaviour using electronic health records. We have proposed a number of suggestions to help overcome these challenges, including the development of new measures, which appreciate the complexity of help-seeking, and acknowledge the reality that help-seeking is rarely a one-off behaviour/event, but rather an ongoing interaction between people and the healthcare system. We have also highlighted gaps in research, where particular populations have so far been underserved in research. As we learn more about where and why inequities exist, and how they are exacerbated, we will be in a better position to deliver robust responses to tackle them.

## Data Availability

No datasets were generated or analysed during the current study.

## Abbreviations

CDC: Centers for Disease Control and Prevention
COVID-19: Coronavirus disease 2019
IMD: Index of multiple deprivation
HQIP: Healthcare Quality Improvement Partnership
NHS: National health service
SES: Socioeconomic status
TRE: Trusted research environment
UK: United Kingdom
